# Regional risks and seasonality in travel-associated campylobacteriosis

**DOI:** 10.1186/1471-2334-4-54

**Published:** 2004-11-29

**Authors:** Karl Ekdahl, Yvonne Andersson

**Affiliations:** 1 Department of Epidemiology, Swedish Institute for Infectious Disease Control (SMI), SE-17182 Solna, Sweden; 2 Department of Medical Epidemiology and Biostatistics, Karolinska Institute, SE-17177 Stockholm, Sweden

## Abstract

**Backgound:**

The epidemiology of travel-associated campylobacteriosis is still largely unclear, and various known risk factors could only explain limited proportions of the recorded cases.

**Methods:**

Using data from 28,704 notifications of travel-associated campylobacteriosis in Sweden 1997 to 2003 and travel patterns of 16,255 Swedish residents with overnight travel abroad in the same years, we analysed risks for travel-associated campylobacteriosis in 19 regions of the world, and looked into the seasonality of the disease in each of these regions.

**Results:**

The highest risk was seen in returning travellers from the Indian subcontinent (1,253/100,000 travellers), and the lowest in travellers from the other Nordic countries (3/100,000 travellers). In Africa, large differences in risk between regions were noted, with 502 /100,000 in travellers from East Africa, compared to 76/100,00 from West Africa and 50/100,000 from Central Africa. A distinct seasonal pattern was seen in all temperate regions with peaks in the summer, while no or less distinct seasonality was seen in tropical regions. In travellers to the tropics, the highest risk was seen in children below the age of six.

**Conclusions:**

Data on infections in returning travellers together with good denominator data could provide comparable data on travel risks in various regions of the world.

## Background

*Campylobacter *infection is a zoonotic disease, observed in most parts of the world. The disease is caused by *Campylobacter jejuni*, or less commonly *Campylobacter coli*. It is estimated to cause 5–14% of diarrhoea, worldwide [1]. The incubation period is usually 2 to 5 days (range 1 to 10 days), and persons not treated with antibiotics may excrete the organisms for as long as 7 weeks [2]. Also in the Western world *Campylobacter *infection has emerged to be most important bacterial cause of gastrointestinal infection. Animals (variety of fowl, swine, cattle, sheep, dogs, cats, and rodents) are the major reservoir for the bacteria. *Campylobacter *does not easily grow in food, but the critical infective dose is low [3].

Unlike salmonellosis with well-known routes of transmission, the epidemiology of campylobacteriosis is still largely unclear, and various known risk factors could only explain limited proportions of the recorded cases [4]. Known risk factors for the disease include ingestion of undercooked meat, contaminated food and water or raw milk, direct contact with pets, farm animals and small children, and swimming in lakes, but also travel abroad [3,5-7]. Direct person-to-person transmission between adults appears to be uncommon. In temperate regions, campylobacteriosis has a distinct seasonal pattern, with the peak incidence in the summer months [4,6,8,9], but seasonal data on campylobacteriosis from tropical regions are scarce.

Approximately 80 million persons from industrialized countries travel every year to places in Africa, Asia, Pacific Islands, Latin America and remote areas of Eastern Europe, and between, 25 and 50 % of travellers to these areas experience travellers' diarrhoea [10-12]. About 80 % of all episodes of traveller's diarrhoea have a bacterial cause, and *Campylobacter *infection is a leading cause together with infections due to enterotoxigenic *Escherichia coli *(ETEC), salmonellosis, and shigellosis [11,13].

In this study we have used returning travellers to Sweden as sentinels to estimate the comparative risks for travel-associated campylobacteriosis in 19 regions of the world, and looked into the seasonality of the disease in each of these regions.

## Methods

### Notification data on campylobacteriosis

Campylobacteriosis is a notifiable disease according to the Swedish communicable disease act. Cases are notified in parallel to the Swedish Institute for Infectious Disease Control (SMI) by the clinician having seen the patient (clinical notification) and the laboratory having diagnosed the pathogen (laboratory notification). At the SMI the notifications from the two sources are merged into case records, using a unique personal identification number issued to all Swedes, and used in all health care contacts. The clinical notifications contain epidemiological information of relevance, including country of infection. For this study we retrieved notification information (age, sex, country of infection and month of infection) from the national surveillance database [14] on all cases of campylobacteriosis notified in the period 1997–2003, with country of infection outside Sweden. All information in the database is derived from the notifications, and the data (including "country of infection") are thus based on the best judgment of the notifying clinician based on the patient history and knowledge of the characteristics of the pathogen in question. Since we focused on travel-associated infections, refugees and newly arrived immigrants (with incomplete personal identification number) were sorted out before analysis.

### Denominator data on travel patterns

Data on travel patterns were obtained from a commercial database, the Swedish Travel and Tourist Database (TDB) [15]. This database contains data from monthly telephone interviews with 2,000 randomly selected Swedish residents, with travel-related questions. Out of the total database, containing data from almost 170,000 interviews, we used 16,255 records of persons with recent overnight travel outside Sweden. Each record included information on principal geographical country/region of travel, age, sex, and month of travel, but no data on any illness. Data from the TDB are often given as regions rather than countries, to account for low numbers of respondents outside the most popular travel destinations.

### Statistical methods

The age, sex and geographical distribution of the interviewees in the TDB, were standardised against the total population of Sweden to give an extrapolation of the actual number of travellers to each country during the seven years. We then estimated risks per 100,000 travellers (divided on the exposures sex, age and region of travel) using notifications on *Campylobacter *infection (cases) as numerator and extrapolated total numbers of travellers from the TDB as denominator. The actual numbers of interviewed persons (controls) were used for the calculations of 95% confidence intervals (95% CI) for the estimates, using the formula:

e^In risk ± 1.96*√ (1/cases+1/controls)^

To adjust for possible confounding and test for interaction, we also calculated odds ratios (OR) with corresponding 95% CI for the same exposures with a logistic regression model.

In an initial crude analysis, odds ratios (ORs) for all exposures (age, sex, and travel destination) on the outcome campylobacteriosis were analysed, with the lowest incidence in each category used as reference. Confounding was then assessed using Mantel-Haenszel stratification. ORs for exposures with significant association with the outcome were included in a logistic regression analysis if they were shown to contribute significantly to the model in a Wald test. The presence of significant interaction was tested with tests for homogeneity. For each region we analysed seasonality separately (OR for disease per month, adjusted for age, sex and number of cases/travellers). All analyses were done using the Stata 6.0 software (Stata Corporation, College Station, Tx, USA).

### Ethical considerations

Notification data is regulated by the Swedish Communicable disease act, and contain full personal identification. The TDB contains aggregated data only. The Medical Ethics Committee of the Karolinska Institute, Stockholm, Sweden, approved the study.

## Results

Of 53,223 persons notified with campylobacteriosis in the period 1997 to 2003, 28,704 (54%) were travel-associated, corresponding to 42.3 cases per 100,000 travellers (Table 1). The total number of infections from single countries both reflected the risk of disease in the various countries, but to a large extent also the travel pattern of Swedes. The five most commonly stated countries of infection were Thailand (n = 6,129), Spain (n = 5,646), Turkey (n = 1,812), Morocco (n = 1,501), and India (n = 1,086).

The 16,255 respondents with overnight travel outside Sweden in 1997–2003 from the TDB database corresponded to almost 68 million travel episodes; 78% leisure trips and 22% business trips (Table 2). Travel to several countries within one region was quite common, but overnight stay in more than one region was rare (less than 0.1% of travellers).

**Figure 1:**
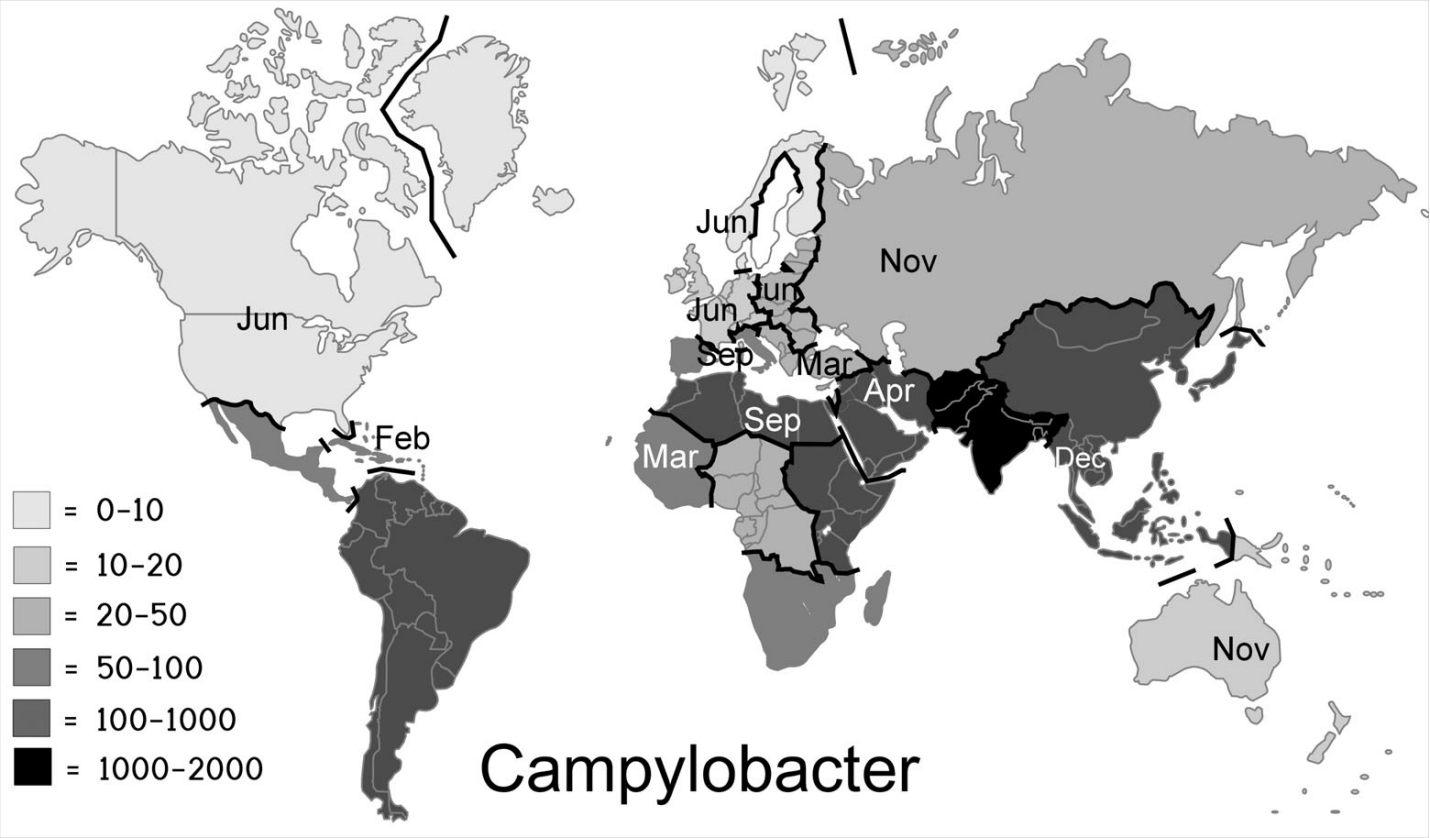
Map showing *Campylobacter *risk per 100 000 returning travellers to Sweden from different regions of the world. In regions with a distinct seasonality, the month with the highest risk (OR) is given.

Comparing the number of cases with the projected number of travellers, we estimated the risk for *Campylobacter *infection in each of the 19 regions under study. The highest unadjusted risks were seen in the Indian Subcontinent (1,253 per 100 000 travellers; 95 % CI 878–1,787), East Africa (502 per 100 000; 95 % CI 314–804), East Asia (386 per 100 000; CI 353–422), North Africa (362 per 100 000; 95 % CI 313–418) and Arab countries/Iran (197 per 100 000; 95 % CI 144–268). Adjusting for age, sex, and month in the logistic regression model did not change the rank between the regions (Tables 1 and 2, Figure 1).

In the crude risk estimate women were at significant higher risk for campylobacteriosis than men; 44.0 cases per 100,000 (95 % CI 42.8–45.2) versus 40.8 cases per 100,000 (95 % CI 39.7–41.9). After adjusting for destination, age, and month in the multivariate logistic regression model, the risks were reversed with a significantly higher OR in males (1.17; 95 % CI 1.11–1.23). However, travel destination was an effect modifier on the association between sex and campylobacteriosis, and this higher risk for males were only significant for travellers returning from a European country (OR 1.21; 95% CI 1.15–1.27) (Table 3).

The highest adjusted age risks were seen in young/middle-aged adults 19–45 years old (OR 2.52; 95 % CI 2.27–2.80) and in small children 0–6 years old (OR 2.34; 95 % CI 1.99–2.76). Also the association between age and campylobacteriosis was modified by travel destination, and in travellers from tropical destinations, especially from Africa and Asia/Oceania the highest risk was seen in the youngest children (Table 3).

There was a marked seasonality in the temperate regions with peak risks mainly in the summer; Nordic countries (peak in June and nadir in March, OR 11.8; 95% CI 5.9–23.4), Western Europe (peak in June and nadir in December, OR 2.4; 95% CI 1.8–3.3), Eastern Europe (peak in June and nadir in November, OR 2.4; 95% CI 1.8–3.3), North America (peak in June and nadir in March, OR 5.8; 95% CI 1.5–23.4), Southern Europe (peak in September and nadir in January, OR 3.9; 95% CI 3.0–5.0), Northern Africa (peak in September and nadir in May, OR 4.3; 95% CI 1.7–11.0), Arab countries and Iran (peak in April and nadir in August, OR 10.1; 95% CI 1.7–26.4), and Australia/New Zealand (peak in November and Nadir in July, OR 33.1; 95% CI 2.8–394). In the Eastern Mediterranean the peak risk was seen in the spring (peak in March and nadir in January, OR 5.1; 95% CI 2.4–10.8), and in Russia and former USSR in late fall (peak in November and nadir in May, OR 6.7; 95% CI 1.3–59.2). In the tropical regions the seasonality was considerably less distinct. In East Asia the risk peak was in December with nadir in May (OR 4.5; 95% CI 2.8–7.2) and in the Caribbean in February with nadir in September (OR 7.8; 95% CI 2.2–27.7). In the Indian Subcontinent, Sub-Saharan Africa, and Central/South America no distinct, significant seasonal peaks could be identified.

## Discussion

### Methodological issues

In this study we report the risks for travel-associated campylobacteriosis and seasonality of the risks in various parts of the world, based on more than 28,000 notified cases. The large number of cases gives more precise risk estimates for this disease than in previous studies, although the estimates are given for quite large regions in parts of the world with few Swedish travellers. The denominator data from the TDB has previously been used in studies on dengue fever [16] and rickettsiosis [17]. We have also tested the reliability of the TDB by comparing the TDB data with in-flight passenger data obtained from some countries with such requirements. For destinations with many travellers, the two sources of information were highly compatible, e.g. less than 5 % difference for travel to Thailand.

Notification data only reflects a small (but unknown) proportion of all travel-related *Campylobacter *infections. One should therefore be cautious in drawing conclusions from the magnitude of the figures, and rather focus on the relative risks between the various regions, as estimated by the odds ratios. Since the data are all from the same source, the risk figures from the various regions are directly comparable. However, there may be a tendency of investigating travellers from the tropics more vigorous than travellers from e.g. the other Nordic countries, thus underestimating the risks in nearby countries. However, such selection bias could likely not explain the huge differences between say West Africa and East Africa.

Since we have no comparable data on the length of stay among the cases in our study, we were not able to include length of stay in our logistic regression model. However, the TDB clearly shows a longer median stay among travellers in far-away destinations. For instance the median stay in Spain was 6 nights, while in Thailand it was 14 nights. On the other hand, only cases detected after the return to Sweden are included in the analysis. The disease data therefore mainly reflect infections contracted during the last week of stay at the travel destination. Differences in length of travel are therefore to some extent evened out.

It has previously been suggested that the risk of travellers' diarrhoea is higher during the first two weeks in highly endemic areas [10,18]. The calculated risks in this study may therefore be underestimated in travel destinations with more prolonged stay. However, persons staying long periods abroad are also less likely to be telephone interviewed in Sweden, balancing the missed cases.

### Regional risks

The differences in risk between various regions were considerable, not only between industrialized and developing countries, but also between different developing countries. The Indian Subcontinent, East Africa, East Asia, and North Africa stood out as special high-risk areas. In a recent Finnish study, the risk of travel associated *Campylobacter jejuni *infection was 10 per 100,000 travellers returning from Spain and Portugal, and 50, 60, and 80 per 100,000 returning travellers from China, Thailand and India, respectively [19]. The lower risks, and lesser differences between the countries may be explained by a much smaller number of cases (n = 205) to base the risk estimates on. East Africa and India have also previously been identified as high-risk areas for travel-associated diarrhoea of various aetiology [20,21], but the very large differences in the risk of campylobacteriosis between East Africa, and West, Central and Southern Africa have to our knowledge not previously been described.

Age, sex and season (month of travel/infection) were identified as possible confounders for the association between travel destination and risk for campylobacteriosis and were thus included in the logistic regression model. All three variables contributed significantly to the model, but the overall effect of these confounders did not alter the rank order between the regions in the logistic regression model compared to the crude analysis.

### Age and gender

The highest risks were seen in young adults and small children, and especially in the tropics the highest risks were seen in the youngest. This is consistent with previous findings that in developed countries the disease most of hits children below the age of 5 years and young adults, while in developing countries it is most often seen in children below the age of 2 years, with an annual incidence of 40–60 % [1]. The data are also consistent with the results from other studies on traveller's diarrhoeas [11]. De Las Casas has suggested that the high risk in young adults is due to a more adventurous lifestyle when it comes to eating habits, and the elevated risk in the youngest is due to increased faecal/oral contamination and decreased immunity [22], explanations that seem plausible. An alternative explanation put forward is that young people with a greater appetite ingest more bacteria, and thereby increasing their risk of infection.

In travellers returning from Europe, male gender was an independent risk factor for *Campylobacter *infection. This pattern is also seen in domestically acquired Swedish campylobacteriosis cases, where 56% of the notified cases in 2003 were males, and in the US where campylobacteriosis is more common in males of all age groups [8,9]. The higher risk in males in the US has been attributed to sex-specific differences in food-handling practices and consumption practices as well as a higher susceptibility to gastro-intestinal infections in males [9]. For travel destinations to tropical or subtropical destinations, the risk was not influenced by gender, consistent with other studies on travel-associated diarrhoea [23].

### Seasonality

In each of the 19 regions in the study, we looked closely at the seasonality of the disease. As has previously been shown [3,6-9], there was a striking seasonal pattern in all temperate regions, with distinct peaks in the summer. Previously these summer peaks have been partly attributed to returning travellers [4], but obviously this could not explain the same peaks in our study. The magnitude of the summer peaks was also in the same order in domestic Swedish cases, as in the returning travellers from other temperate countries.

In the tropical regions, seasonal peaks of campylobacteriosis have not previously been recognized [8]. Also in this study the seasonal pattern was much less distinct in tropical than in temperate regions, and only in East Asia (peak incidence in December) and in the Caribbean (peak incidence in February) could a seasonal pattern be discerned. In a study on US medical students in Mexico, the peak incidence of *Campylobacter *infection was seen between November and April [18]. With only 15 cases and 8 TDB respondents, our study did not have the power to detect any seasonality in Central America.

## Conclusions

Data on infections in returning travellers together with good denominator data could provide comparable data on travel risks in various regions of the world. This study has revealed large and unexplained regional incidence differences, e.g. between East and Central Africa. The very distinct seasonal pattern seen in all temperate regions could not be discerned in the tropics.

## Competing interests

The author(s) declare that they have no competing interests.

## Authors' contributions

KE raised the original idea of the study, did the statistical analyses, and prepared the first draft of the manuscript. YA contributed with in depth knowledge of campylobacteriosis and revised the draft manuscript. Both authors have read and approved the final manuscript.

## Pre-publication history

The pre-publication history for this paper can be accessed here:



## Figures and Tables

**Table 1:** Notified cases of Campylobacter infection in Sweden 1997–2002, per category of infection.

**Disease and category of infection**	**1997**	**1998**	**1999**	**2000**	**2001**	**2002**	**2003**	**1997–2003**
Sweden	1 856	2 616	2 208	2 462	2 839	2 479	2 688	17 148
Travel associated	3 013	3 769	4 564	4 932	4 730	3 914	3 782	28 704
Immigrants/refugees	309	166	167	150	134	111	133	1 170
Unknown	1 703	846	730	870	875	633	544	6 201
Total	6 881	7 397	7 669	8 414	8 578	7 137	7 147	53 223

**Table 2:** Estimated number of Swedish travellers, respondents in the Travel and Tourist database – TDB (controls) and notified Swedish cases with travel associated campylobacteriosis 1997–2003, with an unadjusted risk estimate (per 100,000) and multivariate odds ratios from a logistic regression model adjusted for the risk factors age, sex month of travel and travel destination.

**Age/Sex/Region^a^**	**Estimated no, of travellers**	**Controls (TDB)**	**Notified, cases**	**Risk per 100,000**	**95% CI**	**Multivariable odds ratio**	**95% CI**
Total	67,870,000	16,255	28,704	42.3	41.5–43.1	-	-
0 to 6 years	3,300,000	524	1,234	37.4	33.8–41.4	2.34	1.99–2.76
7 to 18 years	8,150,000	1,599	1,957	24.0	22.5–25.7	1.33	1.17–1.51
19 to 45 years	30,520,000	6,708	16,207	53.1	51.6–54.6	2.52	2.27–2.80
46 to 65 years	21,850,000	5,990	8,200	37.5	36.3–38.8	1.50	1.35–1.66
65+ years	4,050,000	1,434	1,106	27.3	25.2–29.5	Reference	
Men	36,020,000	8,145	14,694	40.8	39.7–41.9	1.17	1.11–1.23
Women	31,850,000	8,110	14,007	44.0	42.8–45.2	Reference	
Nordic countries	22,730,000	5,350	606	2.67	2.45–2.90	Reference	
Western Europe	14,800,000	3,584	2,238	15.1	14.3–15.9	5.58	5.05–6.17
Southern Europe	12,070,000	2,931	6,730	55.8	53.4–58.2	22.2	20.1–24.4
Eastern Europe (incl. Baltic Republics)	3,320,000	818	1,414	42.6	39.1–46.4	14.9	13.2–16.8
Eastern Mediterranean	7,740,000	1,817	3,260	42.1	39.8–44.6	14.5	13.0–16.0
Russia and former USSR	260,000	59	96	36.9	26.7–51.1	14.3	10.2–20.1
Arab countries and Iran	220,000	44	433	197	144–268	92.0	66–128
Indian Subcontinent	120,000	31	1,503	1,253	878–1787	532	369–769
East Asia	2,050,000	517	7,910	386	353–422	173	152–197
Australia, New Zealand and the Pacific	450,000	116	75	16.7	12.5–22.3	6.64	48.9–9.02
North Africa	770,000	196	2,788	362	313–418	164	138–195
West Africa	80,000	22	61	76.3	47–124	28.8	17.4–47.6
East Africa	90,000	18	452	502	314–804	243	151–394
Central Africa	30,000	8	15	50.0	21–118	17.4	7.2–41.8
Southern Africa	170,000	42	150	88.2	63–124	37.2	26.0–53.2
North America	2,170,000	503	77	3.5	2.8–4.5	1.39	1.08–1.80
Central America	170,000	43	148	87.1	62–122	35.0	24.6–49.9
Caribbean	380,000	95	299	78.7	62.5–99.1	33.0	25.7–42.4
South America	250,000	61	449	180	138–235	76.0	57–101

**^a^Nordic countries **= Denmark, Finland, Iceland, Norway; **Western Europe **= Austria, Belgium, France, Germany, Ireland, Luxembourg, The Netherlands, Switzerland, United Kingdom; **Southern Europe **= Italy, Malta, Monaco, Portugal, Spain; **Eastern Europe **= Bulgaria, Czech Republic, Estonia, Hungary, Latvia, Lithuania, Poland, Romania, Slovakia; **Eastern Mediterranean **= Albania, Cyprus, Former Yugoslavia, Greece, Israel, Turkey; **Russia and former USSR **= Armenia, Azerbaijan, Belarus, Georgia, Kazakhstan, Kyrgyzstan, Russia, Tajikistan, Turkmenistan, Ukraine, Uzbekistan; **Arab countries and Iran **= Bahrain, Iraq, Iran, Jordan, Kuwait, Lebanon, Oman, Qatar, Saudi Arabia, Syria, United Arab Emirates, Yemen; **Indian Subcontinent **= Afghanistan, Bangladesh, Bhutan, India, Maldives, Nepal, Pakistan, Sri Lanka; **East Asia **= Brunei, Burma, Cambodia, China, Hong Kong, Indonesia, Japan, Laos, Malaysia, Mongolia, North Korea, Philippines, South Korea, Singapore, Taiwan, Thailand, Tibet, Viet Nam; **Australia, New Zeeland, and the Pacific **= American Samoa, Australia, Cook Islands, Fiji, French Polynesia, Guam, Kiribati, Marshall Islands, Micronesia, Nauru, New Caledonia, New Zealand, Niue, Palau, Papua New Guinea, Samoa, Tokelau, Tonga, Tuvalu, Vanuatu, Wallis and Futuna; **North Africa **= Algeria, Egypt, Libya, Morocco, Tunisia; **West Africa **= Benin, Burkina Faso, Cape Verde, Ghana, Guinea, Guinea-Bissau, Ivory Coast, Liberia, Mali, Mauritania, Senegal, Sierra Leone, The Gambia, Togo; **East Africa **= Burundi, Djibouti, Eritrea, Ethiopia, Kenya, Rwanda, Seychelles, Somalia, Sudan, Tanzania, Uganda; **Central Africa **= Cameron, Central African Republic, Chad, Congo Brazzaville, Equatorial Guinea, Gabon, Niger, Nigeria, Republic of Congo, São Tomé et Principe; **Southern Africa **= Angola; Botswana, Lesotho, Madagascar, Malawi, Mauritius, Mozambique, Namibia, South Africa, Zambia, Zimbabwe; **North America **= Canada, USA; **Central America **= Belize, Costa Rica, El Salvador, Guatemala, Honduras, Mexico, Nicaragua, Panama; **Caribbean **= Antigua and Barbuda, Bahamas, Barbados, Bermuda, Cayman Islands, Cuba, Dominica, Dominican Republic, Grenada, Guadeloupe, Jamaica, Haiti, Martinique, Netherlands Antilles, Puerto Rico, S:t Christopher and Nevis, S:t Lucia / S:t Vincent, Saint Kitts-Nevis, The Grenadines, Trinidad and Tobago, Virgin islands; **South America **= Bolivia, Brazil, Colombia, Ecuador, French Guiana, Guyana, Honduras, Paraguay, Peru, Suriname, Uruguay, Venezuela.

**Table 3:** Multivariable odds ratios (per continent) for the risk of being notified with travel-associated campylobacteriosis from a logistic regression model adjusted for the risk factors age, sex month of travel and travel destination. For North America there were too few cases for any meaningful results.

	**All regions**		**Europe**		**Asia + Oceania**		**Africa**		**Latin America + Carribean**	
**Age/sex**	**OR**	**95% CI**	**OR**	**95% CI**	**OR**	**95% CI**	**OR**	**95% CI**	**OR**	**95% CI**

Men	1.17	1.11–1.23	1.21	1.15–1.28	1.10	0.93–1.29	1.06	0.83–1.37	0.85	0.62–1.17
Women	Reference		Reference		Reference		Reference		Reference	
0 to 6 years	2.34	1.99–2.76	2.00	1.67–2.39	9.39	4.66–18.90	7.67	2.76–21.29	2.89	0.89–9.41
7 to 18 years	1.33	1.17–1.51	1.28	1.11–1.48	1.82	1.16–2.85	1.64	0.91–2.98	1.25	0.47–3.31
19 to 45 years	2.52	2.27–2.80	2.41	2.27–2.80	4.04	2.81–5.84	3.86	2.44–6.10	2.15	1.01–4.56
46 to 65 years	1.50	1.35–1.66	1.45	1.29–1.62	1.99	1.38–2.89	1.74	1.11–2.74	1.41	0.65–3.04
65+ years	Reference		Reference		Reference		Reference		Reference	
